# Allelic Variants of Melanocortin 3 Receptor Gene (MC3R) and Weight Loss in Obesity: A Randomised Trial of Hypo-Energetic High- versus Low-Fat Diets

**DOI:** 10.1371/journal.pone.0019934

**Published:** 2011-06-14

**Authors:** José L. Santos, Rolando De la Cruz, Claus Holst, Katrine Grau, Carolina Naranjo, Alberto Maiz, Arne Astrup, Wim H. M. Saris, Ian MacDonald, Jean-Michel Oppert, Torben Hansen, Oluf Pedersen, Thorkild I. A. Sorensen, J. Alfredo Martinez, NUGENOB Consortium

**Affiliations:** 1 Department of Nutrition, Diabetes and Metabolism, Pontificia Universidad Católica de Chile, Santiago, Chile; 2 Department of Nutrition and Food Sciences, Physiology and Toxicology, University of Navarra, Pamplona, Spain; 3 Department of Public Health and Department of Statistics, Pontificia Universidad Católica de Chile, Santiago, Chile; 4 Centre for Health and Society, Institute of Preventive Medicine, Copenhagen University Hospital, Copenhagen, Denmark; 5 Department of Human Nutrition, Faculty of Life Science, University of Copenhagen, Copenhagen, Denmark; 6 Department of Human Biology, Nutrition and Toxicology Research Institute Maastricht, Maastricht University, Maastricht, The Netherlands; 7 Department of Nutrition, Hotel-Dieu Hospital University Pierre-et-Marie Curie (Paris 6), Human Nutrition Research Center Ile-de-France, Paris, France; 8 School of Biomedical Science, University of Nottingham Medical School, Queen's Medical Centre, Nottingham, United Kingdom; 9 Hagedorn Research Institute, Gentofte, Denmark; 10 Institute of Biomedical Science, University of Copenhagen, Copenhagen, Denmark; Mayo Clinic, United States of America

## Abstract

**Introduction:**

The melanocortin system plays an important role in energy homeostasis. Mice genetically deficient in the melanocortin-3 receptor gene have a normal body weight with increased body fat, mild hypophagia compared to wild-type mice. In humans, Thr6Lys and Val81Ile variants of the melanocortin-3 receptor gene (*MC3R*) have been associated with childhood obesity, higher BMI Z-score and elevated body fat percentage compared to non-carriers. The aim of this study is to assess the association in adults between allelic variants of *MC3R* with weight loss induced by energy-restricted diets.

**Subjects and Methods:**

This research is based on the NUGENOB study, a trial conducted to assess weight loss during a 10-week dietary intervention involving two different hypo-energetic (high-fat and low-fat) diets. A total of 760 obese patients were genotyped for 10 single nucleotide polymorphisms covering the single exon of *MC3R* gene and its flanking regions, including the missense variants Thr6Lys and Val81Ile. Linear mixed models and haplotype-based analysis were carried out to assess the potential association between genetic polymorphisms and differential weight loss, fat mass loss, waist change and resting energy expenditure changes.

**Results:**

No differences in drop-out rate were found by *MC3R* genotypes. The rs6014646 polymorphism was significantly associated with weight loss using co-dominant (p = 0.04) and dominant models (p = 0.03). These p-values were not statistically significant after strict control for multiple testing. Haplotype-based multivariate analysis using permutations showed that rs3827103–rs1543873 (p = 0.06), rs6014646–rs6024730 (p = 0.05) and rs3746619–rs3827103 (p = 0.10) displayed near-statistical significant results in relation to weight loss. No other significant associations or gene*diet interactions were detected for weight loss, fat mass loss, waist change and resting energy expenditure changes.

**Conclusion:**

The study provided overall sufficient evidence to support that there is no major effect of genetic variants of *MC3R* and differential weight loss after a 10-week dietary intervention with hypo-energetic diets in obese Europeans.

## Introduction

Obesity and hyperphagia displayed by animal models with genetic alterations in the leptin-melanocortin system outline the importance of these biological pathways in body weight regulation [1]. Genetic ablation of melanocortin 4 receptor gene in mice produces hyperphagia and obesity [2] while rare mutations in the corresponding human *MC4R* are a cause of severe childhood obesity [3]. Contrary to the effects observed in mice Mc4r gene, the Mc3r−/− mouse shows an unique phenotype characterized by higher percentage of body fat without increased body weight, mild hypophagia, higher energy efficiency, reduced locomotor activity, hyperleptinemia and reduced linear growth compared with the Mc3r+/+ mouse [4], [5]. Indeed, it has been reported that mice with genetic disruption in both Mc3r and Mc4r are heavier than Mc4r−/− mice, indicating a possible non-redundant participation of both melanocortin receptors in obesity and adiposity-related phenotypes [4].

**Figure 1:**
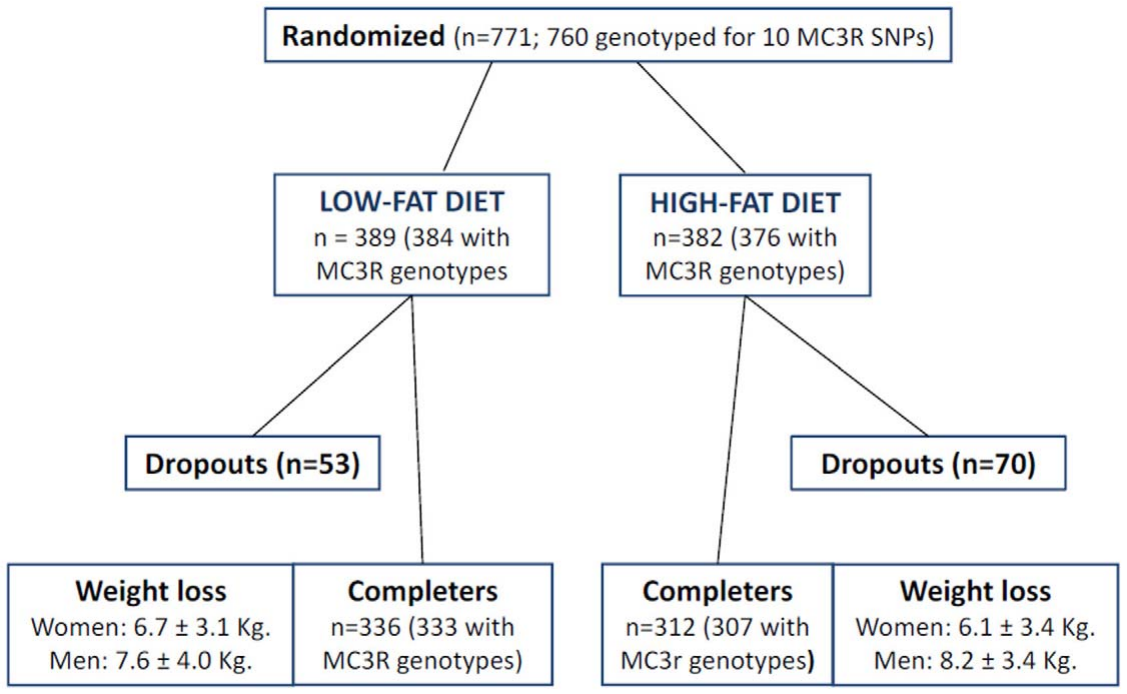
Flow chart of the NUGENOB trial and genotyping success of MC3R variants. Legend to Figure 1: weight loss is expressed as mean ± standard deviation.

Some linkage studies are concordant with the presence of a susceptibility gene for human obesity at the melanocortin-3 receptor *MC3R* locus (gene ID:4159; 20q13.2-13.3) [6], [7]. The missense variants Thr6Lys (rs3746619) and Val81Ile (rs3827103) of *MC3R* have been related with *in vitro* diminished functionality and expression of the receptor, showing a significant association with childhood obesity in a case-control study [8]. Additionally, some mutations in *MC3R* have been proposed as a cause of human monogenic obesity [9]. Genetic epidemiological studies have reported different results regarding the association between common *MC3R* variants and common forms of obesity [10]–[14]. Additionally, large genome-wide association studies have not been able to substantiate associations between variants near the *MC3R* locus and polygenic obesity [15]. In this context, there is published evidence to support a role of the *MC3R* variants Thr6Lys and Val81Ile in weight loss responses of obese children [16]. Herein, we have used previously published data from the NUGENOB study (http://www.nugenob.org) (Figure 1) [17] to assess the association between genetic variants of *MC3R* with weight loss induced by hypo-energetic low-fat/high-fat diets in obese European adults.

## Methods

### Ethics Statement

The selection of participants was carried out in the frame of the NUGENOB project (http://www.nugenob.org) [17], under the approval of Ethical committees at each of the participating centers. Volunteers were informed about the nature of the study, and written consent was obtained prior to study participation. The names of the ethics local committees were: Research Ethic Committee of the University of Navarra, The Danish Research Ethics Committee System, Medical ethics committee of the Maastricht University, Ethics Committee of the Hotel-Dieu Hospital, University of Nottingham Medical School Ethics Committee, The Swedish National Council on Medical Ethics and Ethics Commission of the medical School of Toulouse.

### Study Design and Subjects

The NUGENOB trial consisted in a 10-week dietary intervention with two types of hypo-energetic (−600 kcal/day) diets with a targeted fat energy of 20%–25% (low-fat diet, 15% energy from protein and 60–65 from carbohydrates) or 40%–45% (high-fat diet, with 15% energy from protein and 40–45 from carbohydrates) in obese patients [17]. The study involved 771 European obese adult subjects from eight recruiting centres of seven countries: United Kingdom (Nottingham), the Netherlands (Maastricht), France (Paris and Toulouse), Spain (Pamplona), Czech Republic (Prague), Sweden (Stockholm), and Denmark (Copenhagen). Obese patients participated in this randomised, parallel, two-arm, open-label multi-centre trial to assess weight loss. Obese patients were allocated to the two hypo-energetic diets by stratified block randomization using computer list generated at the coordinating centre.

The participants were 570 women and 192 men, aged 20–50 years and overweight or obese, with a high percentage (94.7%) of subjects with obesity, defined as having Body Mass Index (BMI) greater than or equal to 30 Kg/m^2^ (Median BMI = 34.7 Kg/m^2^; Mean BMI = 35.6 Kg/m^2^; Standard deviation BMI = 4.9 Kg/m^2^). Exclusion criteria were: subjects with changes of more than 3 Kg within the three months previous to the trial, clinically diagnosed hypertension, diabetes or hyperlipidemia treated by drugs, untreated thyroid disease, surgically or drug-treated obesity, pregnancy, or alcohol or drug abuse.

### Dietary Intervention

All the details regarding the dietary intervention are described in Petersen et al. [17] and in the web page http://www.nugenob.org. Before assignment to the dietary intervention group, all participants underwent a thorough standardized clinical and physiological examination described in Standard Operational Procedures applied in all participating centers. The target macronutrient composition of the two diets was: Low-fat (LF) diet (20%–25% of total energy from fat, 15% from protein and 60%–65% from carbohydrate) and High-fat (HF) diet (40%–45% of total energy from fat, 15% from protein, and 40%–45% from carbohydrate). Both diets were designed to provide 600 kcal/d fewer than the individually estimated energy requirements, which was calculated based on the estimation of the measured pre-treatment resting metabolic rate multiplied by a physical activity factor of 1.3. The dietary instructions were designed to minimize possible differences between the two diets in other components such as source and type of fat, amount and type of fiber, type of carbohydrate, amount of fruit and vegetables, and in meal frequency patterns, while taking local customs into account as appropriate. Participants were requested to abstain from alcohol consumption during the trial. The dietary instructions were reinforced and monitored and participants were weighed weekly. Participants were advised to follow their habitual activity patterns throughout the dietary intervention period.

### Anthropometry, Body Composition and Resting Energy Expenditure

Before randomization and after completion of the 10-week intervention, participants underwent a clinical investigation protocol. Measurements of weight and height were carried out with a calibrated set of stadiometers and scales, in light indoor clothes and without shoes. Waist circumference was measured while the participants were wearing only nonrestrictive underwear. The mean of three measurements was recorded for each anthropometric variable. Fat mass was assessed by multifrequency bioimpedance (QuadScan 4000; Bodystat, Isle of Man, British Isles). At the beginning of the intervention, Resting Energy Expenditure (REE) was measured in all participants by indirect calorimetry using open circuit ventilated hood systems and pooled from different centers through a validation program. After the 10-week intervention, REE was again measured in 440 participants (345 women and 95 men) of the individuals who completed the intervention. REE (kcal/24 h.) was calculated with the equation of Weir [18].

### SNP Selection and Genotyping

**Figure 2:**
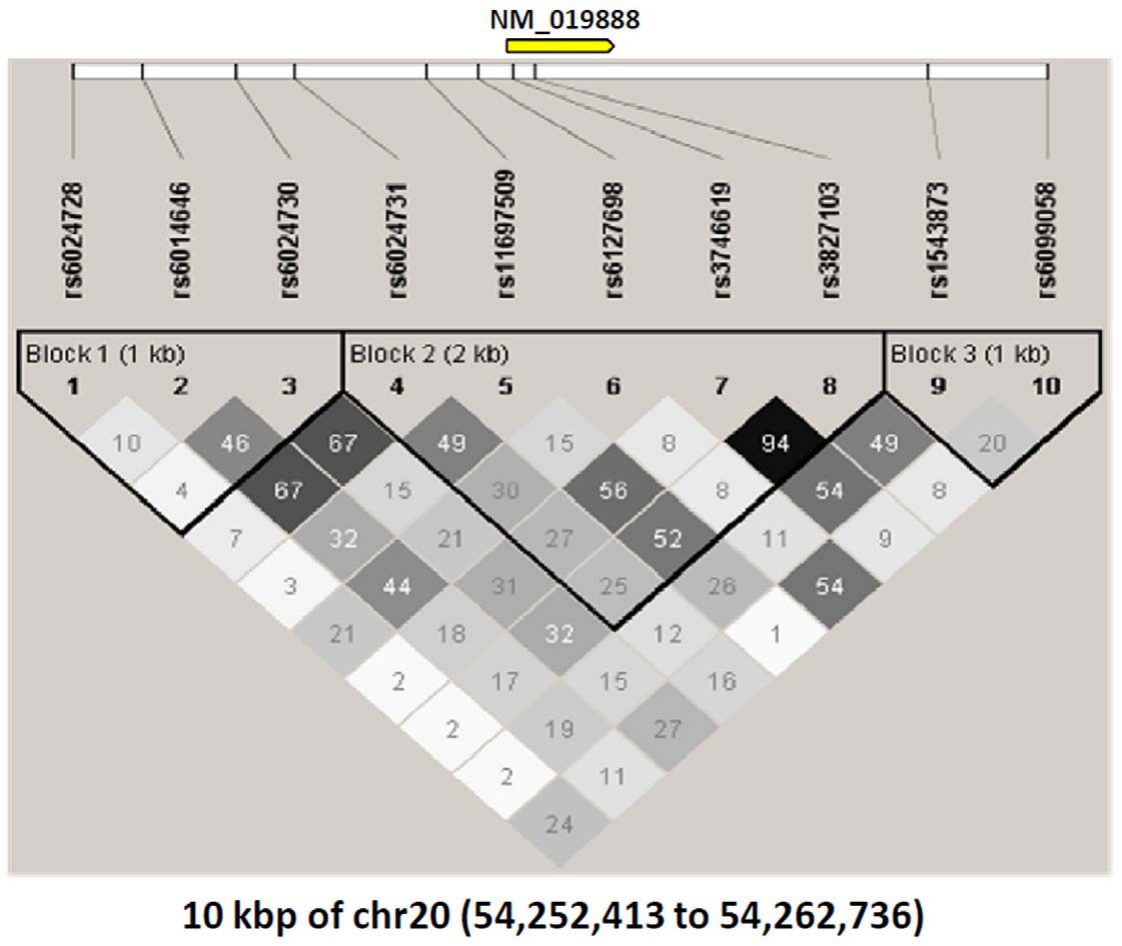
MC3R polymorphisms genotyped in this study and linkage disequilibrium (r^2^) measures.

The single exon of *MC3R* (geneID 4159, 20q13; 1083 base pairs) and its flanking region (5 kb upstream and downstream) was evaluated for the selection of SNPs (MC3R sequences: NC_000020.9; NT_011362.9; NM_019888.2; NP_063941.2). The Tagger program within the HapMap database (http://www.hapmap.org) was used to select the most appropriate set of markers for this study under the criteria of minor allele frequency (MAF) 5% and r^2^<0.8. After incorporating tagSNPs in the coding region (rs3827103 and rs3746619), additional 8 markers were selected: 6 SNPs located 5 kb upstream *MC3R* (rs6024728, rs6014646, rs6024730, rs6024731, rs11697509 and rs6127698) and 2 SNPs located 5 kb downstream of MC3R (rs1543873 and rs6099058) (Figure 2).

Blood samples, drawing and processing, were performed according to international guidelines for genetic studies. Genotyping was carried out by KBiosciences using their TaqMan allelic discrimination assay (http://www.kbiosciences.com). Genotyping success rate ranged from 99.1% (rs6024728) to 99.7% (rs6099058). A total of 760 participants of the NUGENOB trial were genotyped for *MC3R* allelic variants (Figure 1).

### Statistical analyses

Descriptive summary statistics of the variables involved in the study was carried out, including estimation of genotype/allele frequencies and Hardy-Weinberg equilibrium (HWE) assessment. Disequilibrium coefficients r^2^ and D′ were computed as a measure of Linkage Disequilibrium (LD) between pairs of SNPs. Logistic regression analysis was used to assess the association between drop-out rates with genotypes and type of diet. Additional multivariate adjustment (BMI, age, sex and centre) yielded essentially the same conclusion as non-adjusted models.

The outcome variables assessed in this study were weight loss, fat-mass loss, waist circumference changes and REE changes by genotype after the intervention with hypo-caloric diets. Phenotype changes were calculated by subtracting measurements carried out immediately before randomization from measurements performed after the completion of the trial. Linear mixed models were used separately to assess changes for each outcome and for each SNP in *MC3R* gene. Genotype codification was managed using the co-dominant model in which the genotypes were coded as 0, 1 or 2 depending on the number of alternative alleles in the genotype. Additionally, general models (genotypes as dummy variables with the homozygous wild-type as reference) as well as dominant and recessive models were fitted to the data. For weight loss, regression analyses were carried out with type of hypo-energetic diet, gender, age, age^2^, initial weight, gender*initial weight, and centre of study (random variable effect) as covariates. In models for changes in fat mass, waist and REE, we controlled for respective baseline values, an interaction term between gender and initial values for each phenotype, as well as the type of hypo-energetic diet, gender, age, age^2^, and centre of study (random variable effect). Changes in REE were additionally adjusted by fat-free mass and change in fat-free mass. Gene*diet interactions between *MC3R* genotypes and type of diet (low-fat and high-fat) were assessed in the context of co-dominant models for each outcome variable using the multivariate models described above. Haplotype-based association analysis was carried out for weight loss using likelihood ratio tests or permutations. Associations were adjusted by diet, initial weight, age, age^2^, gender and centre of study as covariates. Statistical analyses were carried out with the programs R (v.2.8.1), STATA 11.0, HAPLOVIEW 4.1 and UNPHASED 3.1 [19].

As previously reported for statistical power calculations in the NUGENOB trial [20], the least detectable weight loss of main genetic effects ranged from 1.30–0.78 Kg for dominant models, from 7.72–0.89 Kg for recessive models, and from 1.25–0.55 Kg for additive co-dominant models (allele frequencies ranging from 0.05 to 0.5). Assuming an equal distribution of participants between the diets, the least detectable effects on weight loss of gene-diet interaction ranged from 2.49–1.07 Kg for additive co-dominant models. These calculations were performed with the assumptions of a statistical power of 0.80, a sample size of 642 individuals, and a mean weight loss of 6.8 Kg with a standard deviation of 3.5 Kg (software QUANTO 1.0; http://hydra.usc.edu/gxe).

## Results

Allele/genotype frequencies for *MC3R* polymorphisms and p-values for Hardy-Weinberg equilibrium are shown in Table 1. Very high levels of LD in our sample (Figure 2) were found between the two coding SNPs rs3746619 (Thr6Lys) and rs3827103 (Val81Ile) of *MC3R* (D′ = 0.99; r^2^ = 0.94). No differences were found in genotype frequencies by study centre using a simple chi-square test. Genotype frequencies in the whole sample were concordant with Hardy-Weinberg proportions except for rs6099058 (Table 1). The degree of deviation was considered as acceptable (expected and observed heterozygosity of 0.47 and 0.43 respectively) to keep rs6099058 in the subsequent analyses.

**Table 1:** Basic statistical analysis of *MC3R* variants.

SNP	Position 20q	Minor Allele Frequency	Percentage of Genotyped	P-value HWE	P-value for differences among study centres
**rs6024728**	54252413	0.18	99.1	0.098	0.35
**rs6014646**	54253186	0.32	97.2	0.132	0.10
**rs6024730**	54254196	0.18	98.3	0.158	0.16
**rs6024731**	54254823	0.25	98.6	0.162	0.13
**rs11697509**	54256257	0.14	98.7	0.911	0.26
**rs6127698**	54256823	0.49	99.3	0.482	0.75
**rs3746619**	54257212	0.09	98.9	0.172	0.39
**rs3827103**	54257436	0.08	99.6	0.206	0.50
**rs1543873**	54261736	0.11	99.6	0.298	0.39
**rs6099058**	54263016	0.38	99.7	0.008	0.66

A total of 120 subjects of the successfully genotyped participants (n = 760; Figure 1) failed to complete the 10-week intervention trial. Table 2 shows drop-out percentages according to MC3R genotypes and type of diet. No significant differences in drop-out were detected across MC3R genotypes, and there were no evidences of gene-diet interactions.

**Table 2:** Drop out according to *MC3R* genotypes and type of diet.

	Drop-outPercentage			Per-allele OR(95%CI)	P-value gene*diet interaction
**rs6024728**	**CC**	**CA**	**AA**	1.1 (0.8–1.6)	0.69
High-fat	18.1	18.1	21.1		
Low-fat	12.9	13.5	23.1		
**rs6014646**	**AA**	**AT**	**TT**	1.1 (0.8–1.5)	0.10
High-fat	16.6	17.2	28.8		
Low-fat	14.3	12.8	9.1		
**rs6024730**	**GG**	**GA**	**AA**	1.0 (0.7–1.5)	0.24
High-fat	17.1	20.6	21.7		
Low-fat	14.3	10.5	14.3		
**rs6024731**	**AA**	**AG**	**GG**	1.1 (0.8–1.5)	0.17
High-fat	16.2	18.8	28.1		
Low-fat	14.3	11.1	14.3		
**rs11697509**	**CC**	**CG**	**GG**	1.3 (0.9–1.9)	0.36
High-fat	16.9	21.6	36.4		
Low-fat	13.3	14.3	0		
**rs6127698**	**GG**	**GT**	**TT**	0.8 (0.6–1.1)	0.31
High-fat	22.5	18.6	12.8		
Low-fat	11.2	15.8	10.8		
**rs3746619**	**CC**	**CA**	**AA**	1.3 (0.8–2.0)	0.92
High-fat	17.7	19.3	33.3		
Low-fat	12.8	16.7	0		
**rs3827103**	**GG**	**GA**	**AA**	1.4 (0.9–2.2)	0.77
High-fat	17.7	19.3	37.5		
Low-fat	12.7	18.4	0		
**rs1543873**	**TT**	**TG**	**GG**	1.3 (0.9–1.9)	0.92
High-fat	17.6	19.7	33.3		
Low-fat	12.7	16.2	0		
**rs6099058**	**GG**	**GA**	**AA**	1.2 (0.9–1.5)	0.54
High-fat	15.4	19.7	21.6		
Low-fat	12.7	13.9	13.2		

*Per-allele OR's for drop-outs and *MC3R* genotypes were calculated using logistic regression with the least frequent allele for each SNP coded a 0, 1 or 2 alleles (see statistical methods).

**Figure 3:**
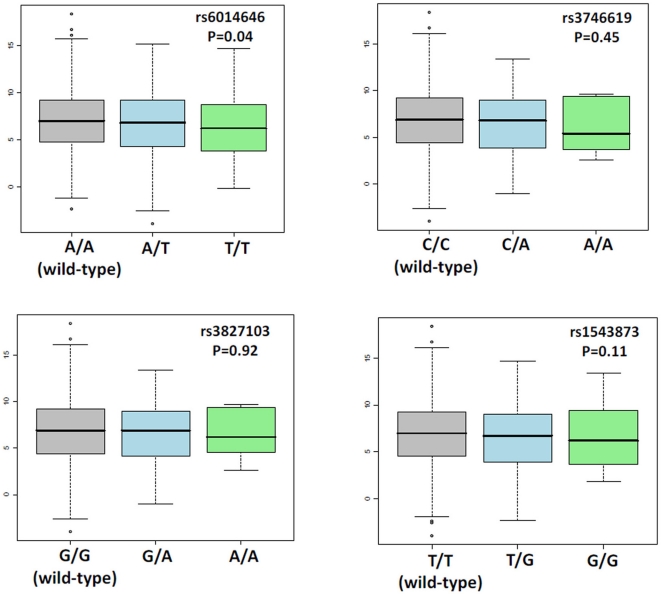
Crude weight loss in the NUGENOB trial by rs6014646, rs3746619 (Thr6Lys), rs3827103 (Val81Ile) and rs1543873 MC3R genotypes. Legend to Figure 3: P-values were calculated from multivariate models (see text).

The overall mean changes of weight, fat mass, waist circumference and REE were −6.8 Kg, −5.3 Kg, −6.3 cm and −109.9 kcal/24 h respectively. Table 3 shows the basic statistical genetic analysis and the main results for *MC3R* genotypes and weight loss and waist change. When assessing weight loss, the T-allele of rs6014646 showed significant association to weight loss using the co-dominant (p = 0.04) and dominant (p = 0.03) models. Using the dominant model, carriers of the T allele in rs6014646 showed −0.55 Kg smaller weight loss (95% CI: −0.03 to −1.06) compared to the AA genotype. The SNP rs6127698 achieved statistical significance level (p = 0.04) to weight loss only in the recessive model. No other significant p-values for association were found at the 0.05 level. Near-significant results were obtained for rs1543873 using the general and dominant models (p = 0.09 and 0.1 respectively). None of the p-values can be considered as significant after controlling for multiple testing. As an example of the magnitude of weight loss and its difference by *MC3R* genotypes, Figure 3 shows crude weight loss by genotypes of the missense variants rs3746619 (Thr6Lys) and rs3827103 (Val81Ile). On the other hand, no gene*diet interaction terms were found to be significant in the multivariate general model when assessing weight loss response in relation to *MC3R* genotypes and the type of diet (low-fat and high-fat) (Table 3). No significant associations or gen-diet interactions were found in assessing changes in waist during intervention. Table 4 shows the main results for *MC3R* genotypes in relation to fat mass loss and REE changes. No statistical genetic effects or gene*diet interactions were detected in this analysis.

**Table 3:** *MC3R* variants, weight loss and waist circumference change in the NUGENOB trial.

	Per-allele* weight loss change (Kg)				Per-allele* waist change (cm)			
SNP	Beta coefficient	Standard error	P-value gene effects	P-value gene*diet interaction	Beta coefficient	Standard error	P-value gene effects	P-value gene*diet interaction
**rs6024728**	0.23	0.23	0.33	0.89	0.01	0.30	0.96	0.47
**rs6014646**	−0.39	0.19	0.04	0.48	−0.29	0.26	0.27	0.84
**rs6024730**	−0.24	0.23	0.30	0.57	−0.18	0.31	0.56	0.39
**rs6024731**	−0.34	0.21	0.10	0.72	−0.27	0.28	0.33	0.76
**rs11697509**	−0.27	0.27	0.33	0.20	0.04	0.37	0.92	0.05
**rs6127698**	0.25	0.18	0.15	0.81	0.23	0.23	0.32	0.62
**rs3746619**	−0.24	0.32	0.45	0.81	0.02	0.43	0.97	0.68
**rs3827103**	−0.03	0.34	0.92	0.90	0.22	0.45	0.63	0.72
**rs1543873**	−0.47	0.29	0.11	0.42	−0.07	0.39	0.85	0.26
**rs6099058**	−0.15	0.18	0.41	0.80	−0.17	0.24	0.48	0.16

*Per-allele changes were calculated using the number of the least frequent alleles for each SNP (coded a 0, 1 or 2 alleles) and under the co-dominant model adjusted for covariates (see statistical methods).

**Table 4:** *MC3R* variants, fat mass and resting energy expenditure (REE) change in the NUGENOB trial.

	Per-allele* fat mass change (Kg)				Per-allele* REE change (kcal/24 h.)			
SNP	Beta coefficient	Standard error	P-value gene effects	P-value gene*diet interaction	Beta coefficient	Standard error	P-value gene effects	P-value gene*diet interaction
**rs6024728**	−0.04	0.22	0.85	0.43	2.08	14.01	0.88	0.09
**rs6014646**	−0.04	0.18	0.84	0.12	−5.18	11.66	0.66	0.09
**rs6024730**	−0.21	0.22	0.34	0.57	−11.47	14.11	0.42	0.25
**rs6024731**	−0.18	0.19	0.35	0.65	−3.25	12.61	0.78	0.06
**rs11697509**	−0.19	0.26	0.46	0.21	−6.95	16.17	0.67	0.05
**rs6127698**	0.04	0.17	0.82	0.85	1.19	10.79	0.91	0.80
**rs3746619**	−0.37	0.30	0.22	0.90	−21.69	19.01	0.25	0.17
**rs3827103**	−0.33	0.31	0.30	0.76	−29.31	20.13	0.15	0.16
**rs1543873**	−0.33	0.27	0.23	0.24	−26.25	17.04	0.12	0.08
**rs6099058**	−0.18	0.17	0.29	0.59	−1.50	10.66	0.89	0.89

*Per-allele changes were calculated using the number of the least frequent alleles for each SNP (coded a 0, 1 or 2 alleles) and under the co-dominant model adjusted for covariates (see statistical methods). Changes in REE were assessed in a subset of the sample (440 participants).

Haplotype-based multivariate analysis for weight loss using sliding-window approach showed that rs3827103–rs1543873 (p = 0.04), rs6014646–rs6024730 (p = 0.06) and rs3746619–rs3827103 (p = 0.10) achieved statistical or near-statistical significant results in relation to weight loss. Permutation test (1000 simulations) yielded p-values of 0.06, 0.05 and 0.10, respectively, for these three haplotypes.

## Discussion

Genotype-dependent response to energy-restricted diets is a relevant topic in personalized nutrition [21], although the genetic effects of known genetic variants on weight loss induced by hypo-energetic diets remain poorly defined [22]. In spite of the importance of the melanocortin system in energy balance [1], [23]–[24], no information was available on the effect of MC3R on weight loss in obese adults submitted to hypo-energetic diets. In a study involving 184 obese children, Santoro et al. [16] found that rs3746619 (Thr6Lys) and rs3827103 (Val81Ile) of *MC3R* were associated with a differential weight loss in response to a negative energy balance in obese children (carriers of 6Lys and 81Ile alleles showed higher resistance to weight loss than non-carriers). We have also found in our study a trend for a greater resistance in losing weight in 6Lys and 81Ile carriers, although without achieving statistical significance. The missense variants Thr6Lys and Val81Ile of *MC3R* are in strong linkage disequilibrium which means that alleles 6Lys and 81Ile frequently occur in the same haplotype [10]–[14], [16]. The simultaneous occurrence of alternative alleles in Thr6Lys and Val81Ile have been also associated in previous studies with childhood obesity, energy intake, eating behavior, substrate oxidation and first-phase insulin secretion [8], [10]–[14], [25].

Research in mice is concordant with a role for Mc3r acting as an inhibitory autoreceptor in Pomc neurons of the arcuate nucleus [26]. According to this role, it has been shown that peripheral administration of selective MC3R agonists stimulates feeding [23], [26]–[28]. However, it is unlikely that this inhibitory action in the arcuate nucleus constitutes the predominant role of Mc3r in energy homeostasis given that this receptor is also expressed in multiple brain nuclei and peripheral tissues [29]. On the other hand, it has been also shown that Mc3r is implicated in the entrainment of food anticipatory behavior [30]. In addition to the possible effect of Mc3r on energy balance, there is also evidence in the literature supporting a different weight gain/loss pattern in Mc3r−/− mice according to the type of diet (high-fat versus low-fat). Butler et al. [28] described that the food intake of Mc3r−/− mice is not increased under low-fat or high-fat feeding in comparison with their wild-type littermates in the context of a specific genetic background. However, when Mc3r−/− mice on a B6 genetic background are fed a high-fat diet, they show hyperphagia and higher body fat when compared with the wild-type mouse [28]. On the other hand, a lower respiratory quotient was observed for Mc3r−/− mice fed with a low-fat diet compared to their wild-type littermates [28]. Finally, fatty acid oxidation in Mc3r−/− mice exposed to a low-fat diet seems to be reduced compared to the oxidation of wild-type mice [31].

Although the NUGENOB trial was primarily designed to study the effect of two diets on weight loss, a secondary goal was the assessment of genetic effects and interactions between diet and genes (NUGENOB stands for “Nutrition, genes and obesity”) [32]. Under this framework, the effect of 42 SNPs in 26 candidate genes were previously assessed in relation to weight loss, finding no significant associations after multiple testing adjustment [20]. Subsequently, an interaction effect was found between the diabetes-associated *TCF7L* genotypes and dietary fat/carbohydrate content [33], and also between *FTO* rs9939609 and macronutrient diet composition [34]. On the other hand, other studies indicated that a common variant genetic variation near *MC4R* gene (rs17782313), a direct candidate gene for food intake and obesity, does not seem to have a relevant effect on weight loss in adults after a lifestyle intervention [35].

Studies in monozygotic twins support the idea that weight loss after negative energy balance is under genetic control [36]. It is important to recognize that the study shown herein can be considered as exploratory, since multiple genetic and environmental factors may influence weight loss after hypo-energetic diets, making difficult the detection of consistent genotype-trait associations [22]. One reason why the main effects are so small could be due to the small magnitude of weight loss and because the study was based on mixed phenotypes with different genetic determinants, such as a combination of general level of obesity with dynamic changes over time. In this context, longitudinal twin studies with repeated assessment of BMI indicate that the genetic influences on general tracking level of BMI is different from those influencing the deviations from the general tracking level, suggesting that the genes influencing the tendency to become obese are different from those inducing individual differences in susceptibility to caloric restriction and dietary changes [37]. The difficulty in finding common genetic variation associated with complex traits is exemplified in the observational studies on increased BMI in large genome-wide epidemiological studies. In these studies common genetic variation seems to explain only a small proportion of BMI z-score in children or BMI in adults [15], [38], with a limited predictive value for obesity risk [39]–[40]. It is conceivable that similar weak effects are present when assessing weight loss after hypo-energetic diets, even using intervention studies with moderately-large sample sizes such as the NUGENOB study.

Genetic variants of *MC3R* have been involved in obesity development mainly in relation with its role in the control of food intake [28]. Given that food intake is restricted in the intervention low-fat and high-fat groups, it was possible that the a priori hypothesized weight loss differences by *MC3R* genotype would have been arisen as a consequence of changes in energy expenditure. However, we have not found significant genetic effects on REE by *MC3R* genotype during the 10-week intervention trial. Regarding the analysis of food intake, it is also possible that assuming allocated diet (high-fat or low-fat) instead of measuring actual diet, has a disturbing effect in finding true associations, due to lack of compliance of participants with the energy deficit imposed by hypo-energetic diets. Regarding drop-out rates, weight loss intervention in the original analysis of the NUGENOB trial showed increased drop-out rates among participants randomized to high-fat compared to low-fat diet [17]. However, we have not found differences in drop-out by *MC3R* genotypes, suggesting that the assessment of the role of *MC3R* variants on weight loss is not affected by this factor. Finally, It is also important to mention that the assessment of gene*diet interactions was hampered by the increased sample size that would have been required to assess such effect in comparison with the sample size needed for evaluating main effects.

As a recapitulation, only *MC3R* rs6014646 achieved nominal statistical significance under the 0.05 level in relation to weight loss in adult obese subjects of the NUGENOB trial. However, this p-value cannot be considered as significant after strict control for multiple testing. Supporting the lack of effect of *MC3R* genotypes on anthropometric variables, tables 1 and 3 show point estimations for the genetic effects on weight loss, fat mass loss, waist and REE changes that are close to the zero effect, with relatively narrow confidence intervals. Moreover, no gene*diet interaction effects were found to be significant. In conclusion, the present study provided overall sufficient evidence to support that there is no major effect of genetic variants of *MC3R* and differential weight loss after a 10-week dietary intervention with hypo-energetic diets in obese Europeans.
